# Rare Localization of Extramammary Paget’s Disease in the Axilla: A Case Report and Literature Review

**DOI:** 10.3390/jcm14238581

**Published:** 2025-12-03

**Authors:** Vera Smolyannikova, Marina Krot, Andrey Filatov, Alina Mordovina, Maria Kharitonova, Olga Gafurova, Valentina Kudryashova, Yulia Lutokhina, Sergey Pirozhkov, Marina Vukolova

**Affiliations:** 1 Institute for Clinical Morphology and Digital Pathology, Sechenov First Moscow State Medical University (Sechenov University), 119991 Moscow, Russialobanova_o_a@staff.sechenov.ru (O.G.);; 2 FGBU State Scientific Center for Dermatovenereology, Cosmetology Clinical Diagnostic Center of the Ministry of Health of the Russian Federation, 107076 Moscow, Russia; maharita@yandex.ru; 3 V.N. Vinogradov Faculty Therapeutic Clinic, Sechenov First Moscow State Medical University (Sechenov University), 119991 Moscow, Russia; lutokhina_yu_a@staff.sechenov.ru; 4 Department of Pathological Physiology, Sechenov First Moscow State Medical University (Sechenov University), 119991 Moscow, Russia; pirozhkov_s_v@staff.sechenov.ru

**Keywords:** extramammary Paget’s disease, axilla, histology, tumor, erythema, inflammation, skin lesion, immunohistochemistry

## Abstract

Extramammary Paget’s disease is a rare malignant tumor that frequently presents in areas rich in apocrine glands, most often affecting the skin of the perianal area: the labia minora, clitoris, scrotum, penis, skin of the lower abdomen, and inguinal folds. Tumors localized outside the perianal region are relatively rare. We present a clinical case of a 70-year-old patient with a tumor localized on the skin of the right axillary region, with clinical, histological, and immunohistochemical characteristics. The diagnosis of extramammary Paget’s disease with atypical localization is relatively difficult to reach in time, as tumors are often mistakenly considered as inflammatory skin lesions, and the appropriate treatment may therefore be delayed.

## 1. Introduction

Extramammary Paget’s disease (EMPD) is a rare malignant tumor first described by G.R. Crocker in 1889 [1]. EMBP often presents in areas rich in apocrine glands, and most often affects the skin of the perianal area: the labia minora, clitoris, scrotum, penis, skin of the lower abdomen, and inguinal folds. Tumors localized outside the perianal region are relatively rare. The literature describes isolated cases of Paget’s disease of the axilla [2], eyelid [3], and ear canal [4]. EMPD may be divided into primary and secondary form; the primary form affects only the skin, while the secondary form comprises the intraepithelial spread of perianal malignancies (adenocarcinomas of the bladder, prostate, and lower gastrointestinal tract).

Data on the incidence of EMPD localized in the perianal region are quite contradictory, ranging from 0.1 to 2.4 per 1,000,000 people [5,6]. This makes up about 1 percent of all tumors with this localization. EMPD is most commonly diagnosed between ages 50 and 80 [7,8], and depending on the site of growth, the peak incidence falls within different age groups. Thus, EMPD of the vulva is most often observed at the age of 50–64 years, while EMPD of the scrotum usually occurs after 70 years of age [9]. EMPD in European women most commonly affects the vulva [10]. Interestingly, the incidence ratio in men and women in Asia is almost 1:1, which may be due to genetic differences compared with the Caucasian population [10].

Only 24 cases of extramammary Paget’s disease of the axilla have been reported in the literature. EMPD of the axilla was diagnosed in patients aged from 40 to 89 years, and included bilateral localization [2,11]. It is noteworthy that in several clinical cases, the occurrence of EMPD of the axilla was combined with sweat gland carcinoma [12,13] and squamous cell skin cancer [14].

Clinically, EMPD often manifests as a slowly enlarging, asymmetrical, red-white scaly plaque that may be painful and itchy [15]. Subsequently, these areas may become erosive, weeping, and crusty. In rare cases, extramammary Paget’s disease manifests as vesicles, vegetative lesions, or regional lymphadenopathy [16]. Itching is the most common symptom; other complaints include burning, irritation and a pain sensation, increased sensitivity, bleeding, or swelling. About 10% of EMPD cases are asymptomatic [17]. Often, EMPD is initially diagnosed as an inflammatory or infectious pathology due to its non-specific clinical signs.

A retrospective study of 246 cases of EMPD in a Chinese male population conducted in 2015 showed a significant time interval between the first manifestations and the final diagnosis in almost all patients, with a mean time of 43.2 months [18]. Interestingly, in one case, it took 30 years to establish a correct diagnosis. In a similar Japanese study that reported the analysis of 145 cases over a time span of 17 years, the mean delay in establishing an accurate diagnosis was 39.7 months [19,20,21,22].

Table 1 presents data about the incidence of EMPD in several countries.

Morphologically, EMPD is characterized by the intraepidermal proliferation of Paget’s cells. There are two main types of Paget’s cells based on their histology: type A, the classical type with porous nuclei, a large number of nucleoli, and pale cytoplasm, and type B, characterized by signet ring cells with a centrally located nucleus and huge droplets of mucin in the cytoplasm [34]. Paget’s cells are mainly located in the lower layers of the epidermis, but single cells are also found at higher levels, such as in the epithelial membranes of hair follicles, excretory ducts of the sebaceous glands, and the secretory part of sweat glands [35]. Large accumulations of Paget’s cells can be found in skin appendages, especially in tangential sections that can sometimes become misinterpreted as invasive tumor growth [36]. The epidermis is usually hyperplastic and displays deep, submerged growths [37]. Suprabasal intraepidermal acantholytic lesions may occasionally be found, and this feature may contribute to the misdiagnosis of EMPD as Darier disease or pemphigus [35]. In addition to traditional microscopy, histochemical methods are also widely used for to establish a proper diagnosis, particularly the PAS reaction for mucin in Paget’s cells. Immunohistochemistry is operational for differentiation between the primary and secondary forms of EMPD, as well as EMPD and various skin tumors [38,39,40,41,42]. Primary EMPD, also known as cutaneous or ectodermal EMPD, is characterized by the expression of sweat gland markers CK7+/CK20-/GCDFP-15+, while secondary EMPD has an endodermal phenotype and is associated with concomitant (primary) carcinoma, most often colorectal (CK7-/CK20+/GCDFP-15-) or urothelial (CK7+/CK20+/GCDFP-15-) [43].

## 2. Methodology

This report includes a description of a clinical case and a review of the literature. The patient selected for studies filled out and signed the informed consent form (ICF). This study was conducted in adherence to the Declaration of Helsinki.

### 2.1. The Review of Literature

The review of the literature was performed using a single database (PubMed) covering the period up to 2025, with no restrictions based on date of publication. The strategy for the literature search and criteria for selection relied on a series of keywords. The following descriptors were used: extramammary Paget’s disease, axilla, histology, tumor, erythema, inflammation, and immunohistochemistry. The authors of this report evaluated the included articles found in the search according to their title and summary and picked out those that fit the inclusion criteria for the study. With a focus on the last 10 years, a pool of reviewed studies was formed, including original articles, reviews, and epidemiological studies. When the reports were scarce on some topics, we also considered earlier publications.

### 2.2. Morphology and Immunohistochemistry

A biopsy of the skin from the edge of the tumor lesion was performed under local anesthesia using a 2% solution of lidocaine hydrochloride. Specimens were fixed in 10% neutral formalin. Formalin-fixed and paraffin-embedded tissue blocks were treated according to the standard procedure. Blocks were used to prepare histological sections stained with H&E. Immunohistochemical studies were performed with the use of the streptavidin–biotin–peroxidase method according to the standard scheme with 5 micron thick sections which were treated using primary monoclonal murine anti-CK7 antibodies (PBM-12F1 clone, Cell Marque, Rocklin, CA, USA). Positive and negative controls were applied in each case with the tissue samples recommended by the manufacturer. The results were evaluated with a light microscope, Leica DM4000B (100×, 200×, 400×). An E3ISPM camera (Suzhou, China) was used for photo fixation.

## 3. Case Report

**Figure 1:**
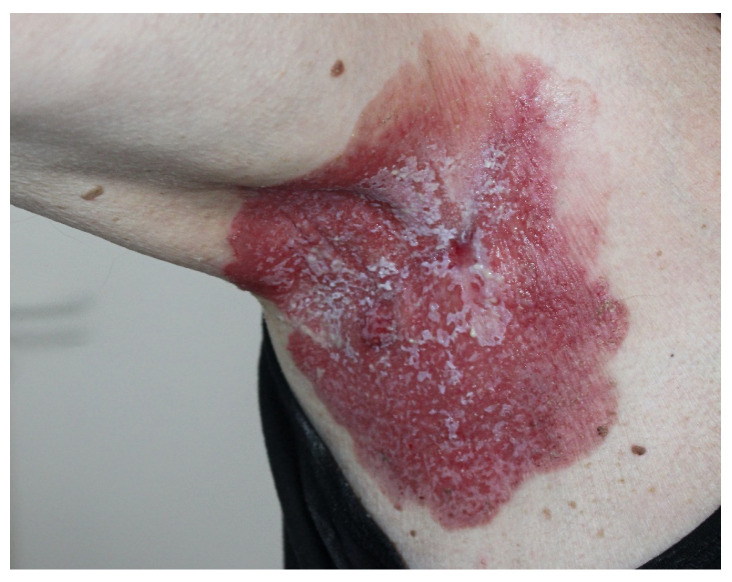
Irregular erythema with clear boundaries with an approximate size of 15 × 18 cm on the skin of the right axillary fold with the transition to the shoulder surface and the lateral surface of the trunk on the same side; there are areas of surface maceration and serous cloudy yellow exudate, occasionally covered with a white coating.

A 70-year-old male patient attended the consultation department of the State Scientific Center for Dermatovenereology and the Cosmetology Clinical Diagnostic Center of the Ministry of Health of the Russian Federation, Moscow, Russia, with complaints of itching and discomfort in the right axillary region. According to his medical history, these symptoms first manifested 15 years ago. The patient noticed redness and maceration of the skin in the axillary area but could not associate it with any specific reason. His primary diagnosis after the first consulting with a dermatologist was “erythematous diaper rash”. The repeated external therapy and courses of antimycotics had no significant effect. Subsequently, the area of the lesion started to enlarge. At the time of examination, the whole skin surface of the right axilla was affected by erythema, which had also extended to adjacent areas. The erythema had clear boundaries, an irregular shape, areas of superficial maceration, and serous dull yellow exudate with a white coating. It was accompanied by an itching sensation (Figure 1). Diagnostic searches included Hailey–Hailey pemphigus, an exudative form of psoriasis, mycosis of large folds, intertriginous dermatitis with the addition of a secondary infection, and pyoderma. A biopsy was performed to clarify the diagnosis.

**Figure 2:**
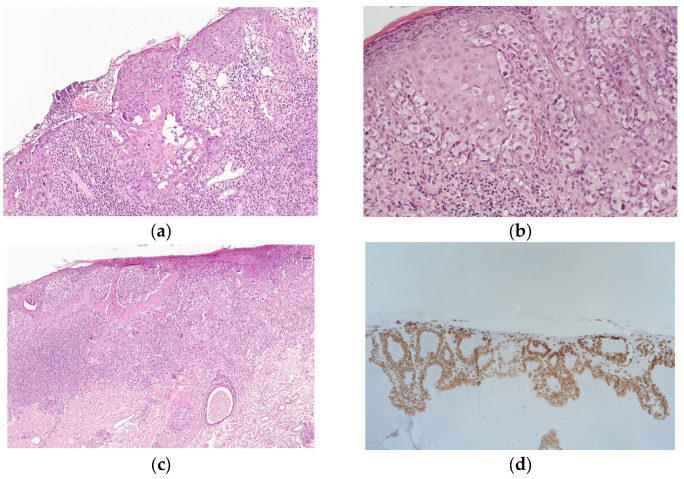
(**a**) Tumor tissue of the epidermis with areas of erosion of the superficial sections throughout (100× magnification); (**b**) tumor cells in the lower layers of the epidermis and lymphohistiocytic infiltrate in the upper part of the dermis (200× magnification); (**c**) the tumor spreads along the excretory ducts of sweat glands and hair follicles (100× magnification); (**d**) diffuse membrane–cytoplasmic expression of CK7 in all tumor cells (100× magnification).

During histological examination, superficial layers of the epidermis were erosive (Figure 2a); the basal and lower layers of the stratum spinosum were replaced by large light cells without intercellular bridges (Figure 2b) spreading along the excretory ducts of sweat glands and hair follicles. In the dermis, there was a moderate lymphocytic infiltrate along the lower border of epidermis (Figure 2c). EMPD was diagnosed on the basis of morphological examination. An immunohistochemical (IHC) study was performed in order to confirm the histogenesis of the tumor using an automatic Bond immunostainer with the antibodies CK7, CK20, p63, and SOX10 on serial sections from the paraffin block using control sections to assess the reaction. Diffuse membrane–cytoplasmic expression of CK7 (Figure 2d) was noted in 100% of tumor cells; the reaction with CK20, p63, and SOX10 was negative. Based on the histological and immunohistochemical studies, a diagnosis of extramammary Paget’s disease was confirmed.

The patient was referred to the cancer center, where the diagnosis of extramammary Paget’s disease was confirmed. Further examination, which included an X-ray of the thoracic cavity and a CT scan with contrasting, revealed the axillary lymph nodes’ involvement in the affected side. Given the size of the skin lesion, it was decided to withdraw from surgical manipulations and to offer radiation therapy to the patient. However, the patient refused to undergo any further treatment.

## 4. Discussion

Extramammary Paget’s disease (EMPD) is a rare malignant tumor that affects the epithelium of apocrine glands. EMPD pathogenesis is still unknown. Based on genomic analysis, somatic mutations have been identified in several genes, including TP53, ERBB, NRAS, BRAF, PIK3CA, and AKT1 [44]. It has been speculated that HER2 encoded by ERBB2 and its signaling pathways, including RAS/RAF-MEK-ERK and PI3K-AKT-mTOR, may play a role in EMPD pathogenesis [45]. In addition, evidence has been obtained that hormonal status may also contribute to the pathogenesis of EMPD. In particular, the increased expression of FOXA1 in Paget cells, noted in some patients with EMPD, has been found to correlate with the expression of estrogen receptors (ER) [46]. Given the fact that FOXA1 supports ER transcriptional activity and that both of these transcription factors may be involved in breast carcinogenesis, FOXA1-ER cooperation may serve as a boosting factor in the development and progression of EMPD [47]. In addition, IHC studies have demonstrated the overexpression of transmembrane prolactin receptor (PRLR) [48,49] and the high expression of androgen receptors (AR), reaching levels of 54–90%. It is remarkable that the age of EMPD manifestation in their studies, as in our report, mostly exceeded 50 years, suggesting the role of dyshormonal disorders as a putative link in the mechanisms of this pathology [50,51,52].

Based on the analysis of the published clinical cases and reports, we have come to the conclusion that the axillary form of EMPD is rare and, as in our case, presents complexities in obtaining the correct diagnosis due to its unusual localization and blurred clinical manifestations. Typically, patients are observed for a long time, with diagnoses varying from eczema to diaper rash, superficial pyoderma, pemphigus, etc. In the reported case, the patient was treated for erythematous diaper rash for 15 years, while the pathological alterations continued to progress. In all cases of the suspected EMPD, its diagnosis can be confirmed only by histological examination. In our observation, the classic form of EMPD was established by a characteristic picture of proliferation of the large light pagetoid cells replacing all layers of the epidermis. The inability of the Paget cells to form intercellular bridges often leads to erosion of the tumor surface, cracks, and blisters, which can be erroneously interpreted as pemphigus, eczema, superficial pyoderma, mycoses, and their variations.

According to the latest recommendations of 2022, the choice of therapy for widespread extramammary Paget’s disease is based on the molecular profile of the tumor and on the presence or absence of metastases [53]. The main direction in the therapy of this disease includes surgical treatment with widespread excision of the tumor. Currently, it is been debated whether there is a need for a 2 to 5 cm indent from the cut-off edges of the tumor, given the possibility of its multicentric growth [54,55]. At the same time, it has been shown in a series of studies that a 1 or 2 cm indent would prove to be sufficient [56]. In cases with a small volume of skin lesions, Mohs micrographic surgery (MMS) may be used.

When surgical removal of the tumor is not recommended, the patient may be suitable for radiation therapy or may be treated via the local application of Imiquimod cream (IMQ) and photodynamic therapy (PDT). The efficiency of radiation therapy and the relapse-free period after treatment are comparable to the results of surgical excision [57,58].

Imiquimod cream (IMQ) is a synthetic ligand of the Toll-like receptor 7 which has been proved to exert an anticancer effect. IMQ efficiency has been demonstrated in therapy of basal cell carcinoma and actinic keratosis. At the same time, monotherapy of extramammary Paget’s disease by IMQ is under debate due to a high proportion of relapses [59]. Treatment of extramammary Paget’s disease by PDT also exhibits moderate efficiency, since it fails to achieve a complete response of the tumor [60,61].

## 5. Conclusions

Our case of extramammary Paget’s disease of the axilla highlights the common difficulties in its diagnosis. It is often misdiagnosed for a long time as an inflammatory skin disease, leading to a significant delay in treatment. Therefore, biopsy is crucial in all patients with eczema and inflammatory skin diseases with an atypical course of pathology manifesting as a large accumulation of apocrine glands and exhibiting no response to treatment within 4–6 weeks. When examining biopsy material, the diagnostic immunohistochemical panel for EMPD is CK7-positive, CK20-positive or -negative, p63-negative, and SOX10-negative [26]. Overexpression of ERBB2 (HER2) [62,63,64] and protein kinase B [65,66,67,68,69,70] may be associated with the invasive forms of EMPD and metastases to lymph nodes [71].

## Figures and Tables

**Table 1:** Case reports of EMPD published in different countries over the past 5 years.

Country	Incidence (Number of Cases Reported in the Literature) References
China	6 [11,23,24,25]
United States	4 [26,27,28]
Japan	4 [29,30,31]
Italy	3 [32]
South Korea	1 [33]

## Data Availability

Data are contained within the article.

## Abbreviations

The following abbreviations are used in this manuscript:

AR	Androgen receptors
EMPD	Extramammary Paget’s disease
ER	Estrogen receptors
ICF	Informed consent form
IHC	Immunohistochemical
PRLR	Transmembrane prolactin receptor
